# Seismic Response Analysis of Steel–Concrete Composite Frame Structures with URSP Connectors

**DOI:** 10.3390/ma15238655

**Published:** 2022-12-05

**Authors:** Linli Duan, Xin Nie, Han Su, Jike Tan

**Affiliations:** 1 School of Civil Engineering, Central South University, Changsha 410075, China; 2 Key Laboratory of Civil Engineering Safety and Durability of China Education Ministry, Department of Civil Engineering, Tsinghua University, Beijing 100084, China; 3 School of Civil Engineering, Beijing Jiaotong University, Beijing 100044, China; 4 State Key Laboratory of Mountain Bridge and Tunnel Engineering, Chongqing Jiaotong University, Chongqing 400074, China

**Keywords:** uplift-restricted and slip-permitted (URSP) connector, steel–concrete composite frame structure, dynamic behavior, finite element, time history analysis

## Abstract

The uplift-restricted and slip-permitted (URSP) connector is a new type of connector used in steel–concrete composite structures that has been proven to improve the structural performance of negative moment regions. Since this connector changes the interface restraint between the slab and steel beam, there is an imperative to study the seismic performance of steel–concrete composite frame systems with this new type of connector. In this study, the dynamic behavior of composite frame structures with URSP connectors under seismic loads was numerically investigated. First, a beam–shell mixed model was used and complex interfaces of different connectors were considered while establishing a numerical model to conduct elasto–plastic time history analysis under various seismic loads. This numerical model was validated with the frame sub-assemblage experimental results of quasi-static cyclic tests. Second, the model analysis results of structures with URSP connectors were obtained and compared with those of traditional structures. Third, dynamic response results including roof displacement, inter-story displacement, and the distribution and failure modes of plastic hinges were analyzed and compared. The comparisons indicated that the arrangement of full-span URSP connectors had a non-negligible influence on the dynamic behavior of the systems. The arrangement increased the maximum inter-story displacement by 31.5% and induced adverse effects in certain cases, which is not suggested in the application of URSP connectors. The partial arrangement of URSP connectors had little influence on the dynamic behavior of the systems, and the frame systems still showed a good seismic performance, which was the same as the traditional composite structural system. These findings may promote the application of URSP connectors in composite structures.

## 1. Introduction

With rapid construction and excellent structural performance, steel–concrete composite structures are widely used in practical engineering [1,2,3]. Since experiments are time-consuming and laborious research methods, many scholars at home and abroad have performed nonlinear response analyses through numerical simulation, which is an important means to grasp the mechanical behavior of composite structural systems under earthquake actions [4,5]. Three types of finite element (FE) models are usually adopted for the elasto–plastic analysis of steel–concrete composite frames: the solid-shell elaborate model [6,7], the beam model [8,9], and the beam–shell mixed model [10,11]. In addition, various models have been adopted to simulate the seismic performance of composite frame systems in recent years, such as a macro-model reflecting slab–column force transfer mechanisms and predicting beam–slab slip demands [12], a continuum FE model reflecting the slab–beam interactions and cyclic degradation of shear stud connectors [13], a 3D FE model in OpenSees [14,15], and a simplified model in SAP2000 [16].

The compatible deformation between steel beams and concrete slabs via shear connectors (spatial composite action) in the elastic and plastic stages significantly impacts the seismic performance of composite frame structural systems. Ignoring the composite action in a simulation may lead to unsafe dynamic response predictions [17,18,19,20,21]. Employing the general finite element software MSC. Marc (2005r2) [22] as a platform, Tao developed a nonlinear analysis subroutine package called COMPONA-MARC [23], which could reasonably consider the influence of spatial composite action on seismic performance and could efficiently conduct the elasto–plastic analysis of steel–concrete composite frame systems under seismic loads. A modified fiber model in OpenSees, which could also accurately and efficiently consider the spatial composite action, was developed by Lin and Zhang [24].

The shear connector is a significant component enabling collaborative work of steel beams and concrete slabs. Some scholars have investigated the influence of the shear connection degree on the dynamic response of composite structures. Zona et al. [25] studied the dynamic behavior of frames with different shear connections under earthquake actions, and they showed that the modal period increased as the shear connection stiffness decreased due to the increase in flexibility. The shear connection degree was found to significantly influence the seismic response of the frame. Brusi et al. [1] studied the nonlinear dynamic response of two four-story frames with full and partial shear connection, and frames with a shear connection degree of about 0.4 performed as well as other frames with a full shear connection under severe seismic loads. Vasdravellis et al. [26] studied the dynamic response of composite frames with different shear degrees and found that partial-strength joints with intermediate and low shear connection degrees were beneficial to seismic design.

**Figure 1:**
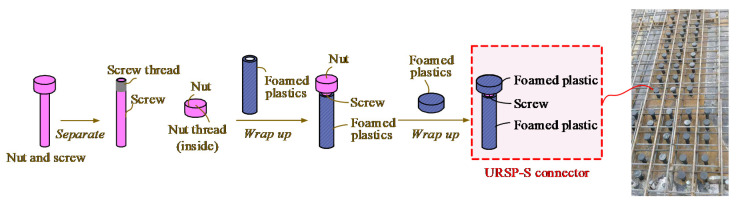
URSP connector.

The uplift-restricted and slip-permitted (URSP) connector, a new type of connector proposed by Nie [27], is used in the negative moment region of composite beams aiming to improve crack resistance performance. This new type of connector is composed of a screw, nut, and low elastic modulus material, as shown in Figure 1. The longitudinal shear resistance of the interface between the steel beam and concrete slab is released due to the low elastic modulus material (foamed plastic); thus, the cracking resistance of the composite structure is improved [28]. The theoretical mechanism of partially releasing the composite effect of the steel–concrete composite beams by applying URSP connectors was explored [29], theoretical models of the uplift and slip performance of URSP connectors were studied [30,31], steel–concrete composite beams with URSP connectors were experimentally studied [32], and curved composite beams with URSP connectors were experimentally and numerically studied [33]. Except for composite beams, investigations of the application of the new type of connector have also included components of composite bridges [34,35], sub-assemblages, and connections of the composite frames of building engineering [36,37,38]. These studies and engineering applications [28] have proven that this novel composite structure with URSP connectors demonstrates good performance and a high crack resistance.

However, the abovementioned research on URSP connectors was mainly focused on the structural performance of the composite component, connections and sub-assemblages, and the seismic performance of steel–concrete composite frame systems with URSP connectors has not been investigated yet. The behavior of a frame system under a seismic load depends on multiple factors, such as the interaction of each component and the dynamic characteristics of the whole structure. After URSP connectors are applied to the beams of a frame, the constraints between the steel beams and concrete slabs are partially released and the spatial composite action is different from structures with traditional shear connectors. Therefore, the response of a composite frame system with the application of URSP connectors under horizontal earthquake actions must be studied. This paper presents the numerical analysis of composite frame systems with URSP connectors under earthquake actions. First, a beam–shell mixed modeling strategy was presented and validated with a test. Then, model and time history analyses were conducted using steel–concrete composite frame system models considering different interfaces. Finally, the roof displacement, inter-story displacement, and plastic hinges induced by earthquakes were analyzed.

## 2. Numerical Model and Validation

The numerical modeling strategy is introduced and validated with test results in this section. These results were employed to conduct the seismic elasto–plastic behavior analysis presented in the next section.

### 2.1. Finite Element Model

For the beam–shell mixed model, multi-layer shell elements are adopted to simulate concrete slabs, fiber beam elements are employed to simulate steel beams, and links are used to simulate connectors between steel beams and slabs. Hence, the effects of different shear connectors and the slip can be considered in this model. When analyzing multi-story and high-rise structures, the complexity of the beam–shell mixed model lies between the solid-shell elaborate model and the beam model, so its efficiency is acceptable. Therefore, the beam–shell mixed model was employed in the follow-up simulation analysis.

**Figure 2:**
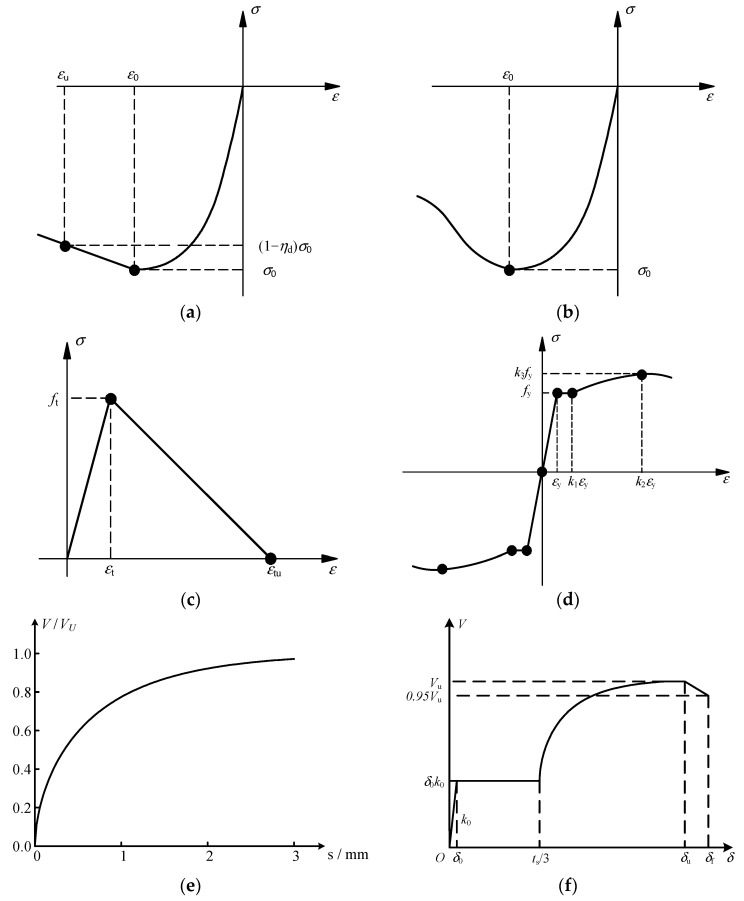
Constitutive curves [23,36,39]: (**a**) ordinary concrete under compression; (**b**) cube concrete under compression; (**c**) concrete under tension; (**d**) steel and reinforcement under tension and compression; (**e**) stud; (**f**) URSP connectors.

The FE model was established through the large-scale general purpose FE program MSC.MARC (r2015) [22]. The fiber beam element proposed by Tao [23,39] can be applied for both steel beam elements and column elements. This fiber beam element can be used for not only composite member elements but also ordinary concrete elements and ordinary steel structural elements, as has been verified by substantial tests. Constitutive curves of steel, reinforcement and concrete are shown in Figure 2a–d with reference to [23,39]. The constitutive relationships of steel and reinforcement were established considering the Bauschinger effect under hysteretic loads, and the constitutive relationship of concrete was established considering the degradation behavior under reciprocating loads, which can accurately simulate the mechanical properties of various materials under seismic loads. The constitutive relationships of the stud connector and the URSP connector are given in Figure 2e,f, whose details were derived from [36]. Equations (1)–(4) describe the shear force–slip relationship curve of URSP connectors, which was proposed and validated by the authors’ research team [36].

(1) $$V=\{\begin{array}{cc} k_{0}\delta & \delta \leq \delta_{0}\\ k_{0}\delta_{0} & \delta_{0}<\delta \leq \frac{t_{s}}{3}\\ V_{u}{\left[1-e^{-\left(\delta -\frac{t_{s}}{3}\right)}\right]}^{0.558}+k_{0}\delta_{0}\leq V_{u} & \frac{t_{s}}{3}<\delta \leq \delta_{u}\\ V_{u}\left[1-\frac{\delta -\delta_{u}}{10\left(\delta_{f}-\delta_{u}\right)}\right] & \delta_{u}<\delta <\delta_{f} \end{array}$$

(2) $$V_{u}=0.43A_{s}\sqrt{E_{c}f_{c}}\leq 0.7A_{s}\gamma f_{s}$$

(3) $$\frac{\delta_{u}}{d_{s}}=0.41-0.0030f_{c}+\frac{t_{s}}{3d_{s}}$$

(4) $$\frac{\delta_{f}}{d_{s}}=0.45-0.0021f_{c}+\frac{t_{s}}{3d_{s}}$$

where *V* is the shear force; *k*_0_ is the elastic stiffness before the interfacial bond is destroyed; *δ* and *δ*_0_ are the interface slip and the slip when the interfacial bond is destroyed, respectively; *δ_u_* and *δ_f_* are the slips at maximum capacity and failure, respectively; *t_s_* is the foamed plastic thickness; *A_s_* is the screw area; *d_s_* is the screw diameter; *V_u_* is the ultimate shear capacity; *E_c_* and *f_c_* are the moduli of the elasticity and compressive strength of concrete, respectively; and *f_s_* is the yield strength of the screw.

### 2.2. Validation by Pseudo-Static Frame Experiments

**Figure 3:**
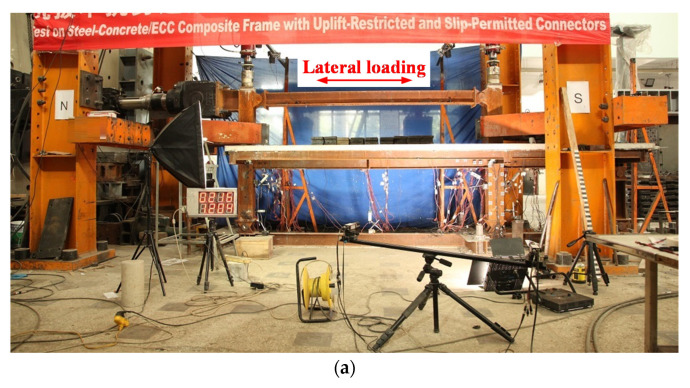

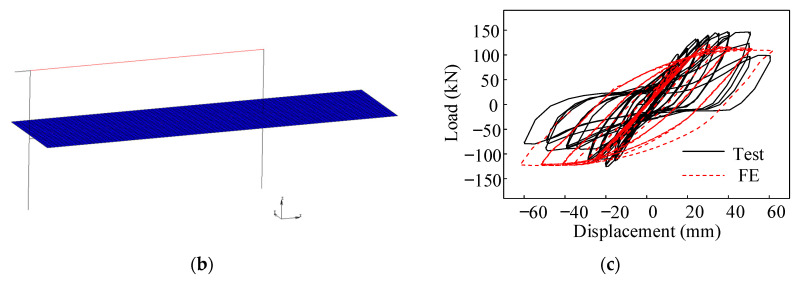
The validation of the FE model with test results: (**a**) test photo of the composite frame with URSP connectors [37]; (**b**) FE model of the test specimen; (**c**) comparison of test and FE model load–displacement curves.

The frame specimen, which consisted of rectangular concrete-filled steel tubular (CFST) columns and steel–concrete composite beams with URSP connectors, was previously constructed and tested under vertical and lateral loads by the authors [37]. Figure 3a presents a test photo of the specimen; the specimen had a length of 4500 mm and a height of 1550 mm, the slab had a width of 1166 mm and a thickness of 60 mm, the CFST column had dimensions of 176 × 166 × 5 mm, and the size of the primary steel beam was 200 × 100 × 6 × 4 mm. URSP connectors were arranged along the whole composite beam. Detailed information on the specimen can be found in [37]. The previously introduced FE modeling method was employed to establish the FE model of the test specimen (shown in Figure 3b). Lateral reciprocating displacements were exerted on the specimen in the FE simulation, and the obtained results were compared with the test results, as shown in Figure 3c. It can be seen that the load–displacement curve of the FE simulation of the test frame agreed well with the test results in terms of the capacity in the negative loading direction, which proved the feasibility of the FE modeling method. For the positive loading direction, the capacity of the FE model was slightly less than the test results, indicating the conservative prediction of the FE model. In general, the average error of bearing capacity in the positive and negative direction did not exceed 10%, which verified the feasibility of the model.

## 3. Seismic Elasto–Plastic Behavior Analysis

### 3.1. Basic Information of the Model

**Figure 4:**
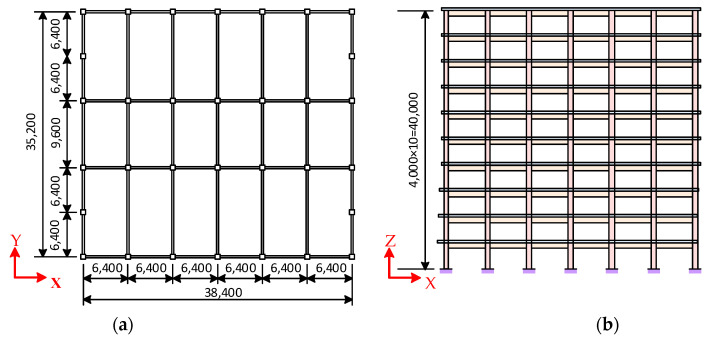
The layout of the structure [40] (unit: mm): (**a**) plan layout; (**b**) elevation layout.

**Figure 5:**
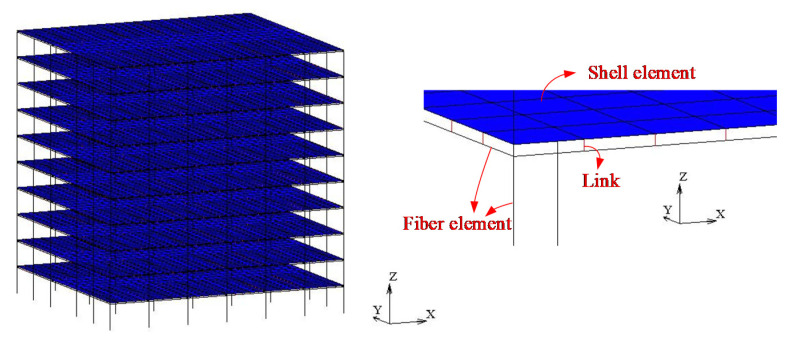
FE model of the investigated structure in MSC.Marc.

The structural system of the 10-story steel–concrete composite frame in [40] shown in Figure 4 was analyzed. The composite frame used a square CFST column with a section side length of 600 mm. The thicknesses of the steel tube of the column were 20 mm (1st–2nd floors), 18 mm (3rd–6th floors), and 16 mm (7th–10th floors). The steel beam was a welded I-shaped steel beam, and the dimensions of the steel beam sections of all floors were 750 × 300 × 14 × 24 mm (beam height × flange width × web width × flange thickness, respectively). The thickness of the concrete floor was 110 mm, and the longitudinal and transverse reinforcement ratios were both 1%. The shear connectors were designed as full shear connectors in accordance with the JGJ 138-2016 “code for design of composite structures” [41]. The concrete degrade of column and floor were C40 and C30, respectively (corresponding to the design values of the axial compression strength of 19.1 N/mm^2^ and 14.3 N/mm^2^, respectively) [42], the steel was Q345, the floor concrete was C30, and the strength grade of the reinforcement was HRB235. The damping ratio of the structure was set as 3.5%. The dead and live loads of the floor were set as 5 kN/m^2^ and 3 kN/m^2^, respectively. When calculating the elasto–plastic time history of the structure under seismic actions, the self-weight of the structure was considered and the material strength was taken as the standard value corresponding to the strength grade. Based on the modeling method described in the previous section, the beam–shell mixed FE model of the 10-story composite frame was established in MSC.Marc, as shown in Figure 5.

### 3.2. Model Analysis

**Figure 6:**
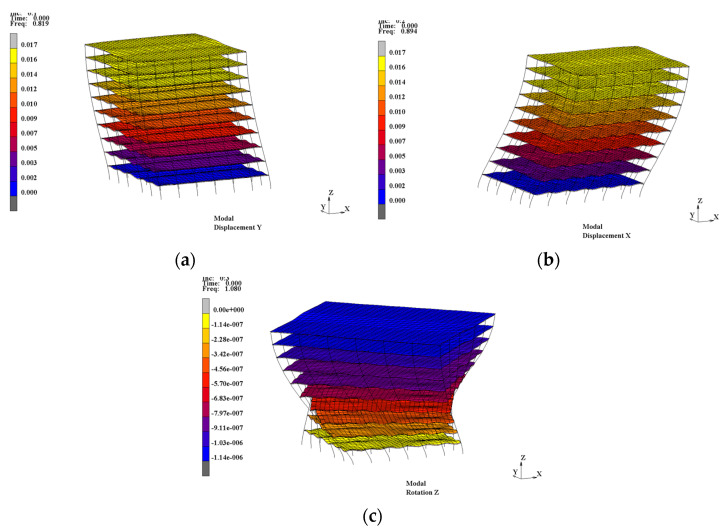
Mode characteristic of the first three modes (full-span URSP connectors): (**a**) 1st mode; (**b**) 2nd mode; (**c**) 3rd mode.

The influence of different interface connections between steel and concrete on the natural vibration characteristics of composite frame structures was investigated. Various cases, including perfect interaction between the steel beam and concrete slab (common nodes), the full-span arrangement of traditional stud connectors (full-span stud), the full-span arrangement of URSP connectors (full-span URSP), no interactions (spring stiffness = 0), and bare steel beam, were considered in the simulation. The natural vibration characteristic results of these various cases were obtained, tabulated and compared. Table 1 shows the first nine calculated natural vibration periods, natural vibration period ratios (full-span URSP/full-span stud), and modal characteristics of each period. The table shows that the natural vibration periods calculated by arranging full-span studs were larger than those with common nodes, indicating that the stiffness of the structure of the former case was smaller, mainly because slip existed in the steel–concrete interface of the traditional stud connection arrangement. Compared with the full-span stud, the natural vibration periods calculated by arranging full-span URSP connectors slightly increased, indicating that the stiffness slightly decreased following the arrangement of the URSP connectors. The restraint between slab and steel beam was released due to the use of the low elastic modulus material of the URSP connectors. Nevertheless, the results of the full-span URSP connectors were different from the results when the interface connection was completely released (spring stiffness = 0), as the natural vibration period reduction in the latter was more obvious. The difference indicated that the interface restrain was not completely released with the application of the full-span URSP connectors, which was reasonable given the actual stiffness of the URSP connectors on the basis of the constitutive curve (seen in Figure 2f). Furthermore, the modal characteristics of the bare steel beam were also calculated; compared with the results of the composite beams with various interface connections, the natural vibration period was greatly increased and the stiffness was greatly reduced. The relationship of stiffness obtained with this model was the same as those obtained with the local node analysis of the elaborate finite element model in [36] and the first-order natural vibration period obtained by the model established with Midas/gen 2015 software in [43]. Figure 6 shows the first three mode shapes of the full-span URSP connectors’ model, with modal properties of Y-translation, X-translation, and Z-rotation. The main mode characteristic was not changed after applying the URSP connectors.

As listed in Table 1, the results showed that the natural vibration periods of the composite frame with the full-span URSP connectors were slightly increased compared with the full-span stud connection, and the differences in natural vibration periods were insignificant. However, it is worth noting that according to the results of pseudo-static frame experiments [37], structural stiffness is significantly decreased with the full-span arrangement of URSP connectors compared with full-span stud connections. Therefore, it is not recommended to arrange full-span URSP connectors in composite structures in practical engineering. Nevertheless, as an ultimate working condition, a case of the arrangement of full-span URSP connectors was used to study the influence of the URSP connector on the elasto–plastic time history response of the structure in further analyses.

### 3.3. Time History Analysis

**Figure 7:**
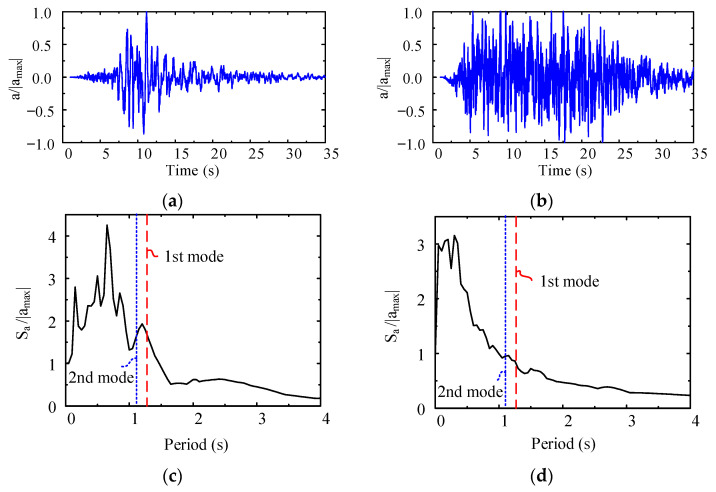
Time–history curves and seismic response spectra: (**a**) time–history curve of seismic wave 1; (**b**) time–history curve of seismic wave 2; (**c**) seismic response spectrum of seismic wave 1; (**d**) seismic response spectrum of seismic wave 2.

Two typical seismic waves were adopted in the time history analysis. Time history wave 1 was the Kobe seismic wave measured on 16 January 1995, and time history wave 2 was a seismic wave in SAP2000 software with a duration of 35 seconds. Figure 7a,b shows the normalized seismic wave time histories according to the acceleration amplitude |a_max_| of two seismic waves, and Figure 7c,d shows the seismic wave response spectra of the single degree of freedom system with a damping ratio of 3.5% of two seismic waves; in the figure, S_A_ is the maximum structural acceleration response corresponding to different structural periods. According to the analysis described in the previous section, compared with the results of full-span stud connection, the natural vibration periods calculated by arranging full-span URSP connectors slightly increased and the stiffness slightly decreased. The first-order period (Y-translation) and the second-order period (X-translation) of the traditional stud connector model are expressed in Figure 7c,d. Near the first-order or second-order periods, with the natural vibration periods slightly increasing, the maximum structural acceleration response could increase or decrease. Therefore, in comparison with the full-span stud, after using the full-span URSP connectors, the differences in the dynamic response results were composed of two parts: a change in the maximum structural acceleration response caused by the increase in the natural vibration period and a reduction in structural stiffness. Due to the superimposition of these two influencing factors, the effect of the URSP connectors on the elasto–plastic analysis results still needed further analysis.

The seismic inputs of the investigated frame included the X and Y directions, 8-degree large earthquake (peak acceleration of 400 gals), and 8-degree small earthquake (peak acceleration of 70 gals). Specifically, four cases—case XL1 (the large earthquake of time–history wave 1 in the X-direction), case YL1 (the large earthquake of time–history wave 1 in the Y-direction), case YS1 (the small earthquake of time–history wave 1 in the Y-direction), and case XL2 (the large earthquake of time–history wave 2 in the X-direction)—were considered, the elasto–plastic time histories in different cases were calculated, and the influence of the application of URSP connectors on the seismic performance of the composite frame was further studied. The simulation results were compared and discussed regarding aspects of roof displacement, inter-story displacement, and the distribution and failure modes of plastic hinges.

## 4. Results, Analysis and Discussion

### 4.1. Roof Displacement

**Figure 8:**
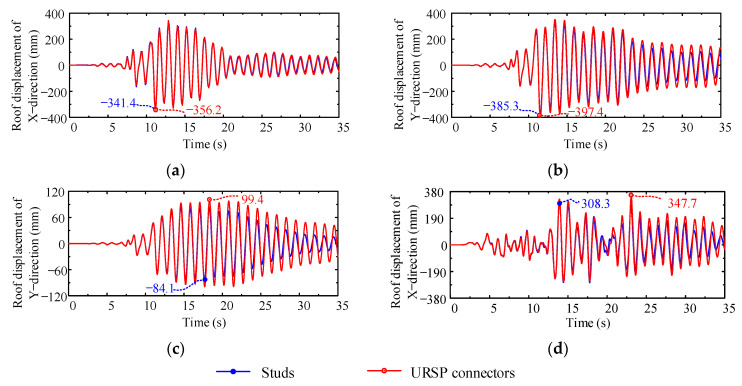
Roof displacement: (**a**) case XL1; (**b**) case YL1; (**c**) case YS1; (**d**) case XL2.

Figure 8 illustrates a comparison of roof displacements in various cases. It can be seen that the maximum roof displacement of the URSP connectors was larger than that of traditional stud connectors. In other words, the maximum roof displacement of a structure with URSP connectors will be underestimated if the effect of the connectors is ignored. The ratios of the calculation results of the URSP connection to the calculation results of traditional stud connection of various cases are shown in Table 2. In cases XL1 and YL1, the difference in the maximum roof displacement of the structure calculated with the two connector arrangements was very small. However, in cases YS1 and XL2, in comparison with the results of the traditional stud connection, the maximum roof displacements of the structure with full-span URSP connectors significantly increased by 18.3% and 12.8%, respectively. Since the full-span arrangement of URSP connectors is an extreme scenario, which is usually not recommended in practice engineering, for case YS1 (with the more obvious difference), the half-span arrangement of URSP connectors was analyzed. It is found that the results were almost the same as those for a traditional stud arrangement, as shown in Table 2. Therefore, the partial arrangement of URSP connectors had little influence on the roof displacement of the structure.

**Figure 9:**
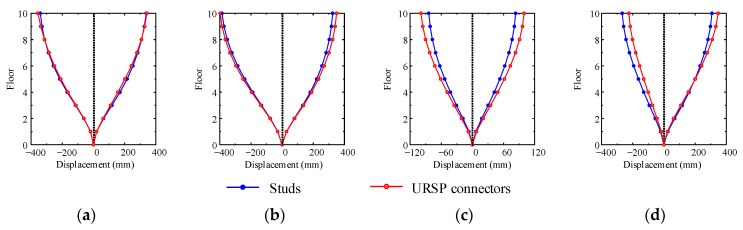
Lateral displacement when the maximum roof displacement occurred: (**a**) case XL1; (**b**) case YL1; (**c**) case YS1; (**d**) case XL2.

**Figure 10:**
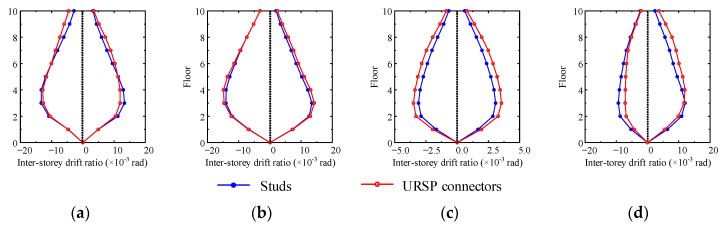
Inter-story drift ratio when the maximum roof displacement occurred: (**a**) case XL1; (**b**) case YL1; (**c**) case YS1; (**d**) case XL2.

Figure 9 and Figure 10 show the corresponding lateral displacements along the floor and the distribution of inter-story drift ratio when the maximum roof displacement occurred, respectively. Under seismic waves, the structure showed characteristics of overall shear deformation. Due to the large axial force of the bottom floor column and the significant *p*-△ effect, strictly controlling the inter-story drift ratio of the bottom floor was found to be of great significance in avoiding overall collapse. If the inter-story drift ratio of the bottom floor increased, it would be unfavorable to the seismic safety of the structure. In cases XL1 and YL1, the use of URSP connectors had little effect on the lateral displacement and inter-story drift ratio, while in cases YS1 and XL2, the lateral displacement and inter-story drift ratio were either increased or decreased after the URSP connectors were arranged.

### 4.2. Inter-Story Displacement

**Figure 11:**
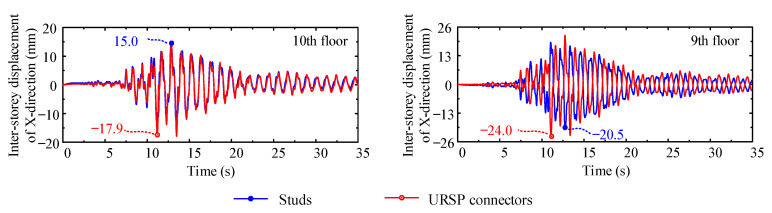
Inter-story displacement time history of case XL1.

In the performance-based seismic design of structures, the inter-story drift ratios must meet the limits specified by the relevant code [44]. The inter-story displacement time histories of each floor of the composite frame with traditional studs and URSP connectors in various cases were obtained, and the partial results of case XL1 are presented in Figure 11. Table 3 shows the maximum inter-story displacements of traditional studs and URSP connectors in various cases. It can be seen from the figure and table that, compared with traditional stud connection, the amplitude of each floor and the time to reach the amplitude of the inter-story drift ratio were slightly different after the URSP connectors were arranged.

To further investigate the influence of the URSP connectors on the maximum inter-story displacement, the ratios of the results of the URSP connectors in various cases to the results of the traditional stud connectors were obtained, as listed in Table 4. Compared with the traditional stud connector arrangement, after the URSP connectors were applied in the full span arrangement in cases XL1 and XL2, the amplitudes of the inter-story displacement of a few floors slightly increased and the amplitudes of the inter-story displacement of most floors were barely changed. In case YL1, the application of full-span URSP connectors had little effect on the displacement amplitude of each floor of the composite frame. However, in case YS1, the arrangement of full-span URSP connectors significantly influenced the displacement amplitude of each floor of the composite frame (the maximum value of the difference was 31.5%). Therefore, after full-span URSP connectors are applied to a structure, if they are designed in reference to the design with traditional studs, there will be a large error in the prediction of this key index, which should be paid attention to.

However, in practical engineering, it is generally not recommended to use full-span URSP connectors due to their reduced stiffness, as established by the results of pseudo-static frame experiments [37]. Accordingly, the response of the composite frame system with half-span URSP connectors in case YS1 is presented below, and the results are tabulated in Table 4. In comparison with the results of the traditional studs, the displacement of each floor was very close and the half-span URSP connectors had little influence on the displacement when the structure was under seismic loads. These results are in line with previous research showing that the partial use of URSP connectors hardly affects the bearing capacity of a structure under static and pseudo-static loads in comparison with a traditional stud connection [32,36,37]. Therefore, the nonlinear analysis subroutine package COMPONASP-MARC, developed by Tao to simulate the earthquake elasto–plastic behavior of composite structures [23], could also be employed for the seismic performance assessment of composite frames with URSP connectors.

**Figure 12:**
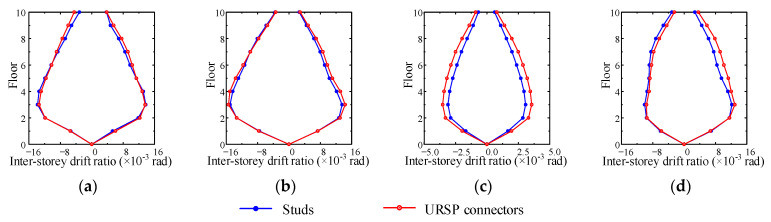
Inter-story drift ratio envelopes: (**a**) case XL1; (**b**) case YL1; (**c**) case YS1; (**d**) case XL2.

Figure 12 shows the envelopes of inter-story drift ratios in various cases. For most cases, the maximum inter-story drift ratios obtained by arranging full-span URSP connectors exceeded the results obtained by arranging traditional stud connectors. Therefore, for the full-span URSP connection composite frame system, even though the maximum inter-story drift ratio satisfied the provisions of the current code [44], it may exceed the actual limit of a frame system in practical engineering and cause insecurity. For the half-span URSP connection composite frame system, as previously illustrated, the URSP connectors had little effect on the inter-story displacement amplitude. Therefore, the influence of URSP connectors on the maximum inter-story drift ratio could also be ignored.

### 4.3. Distribution and Failure Modes of Plastic Hinges

**Figure 13:**
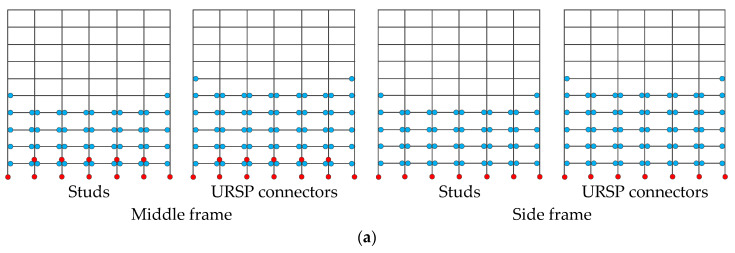

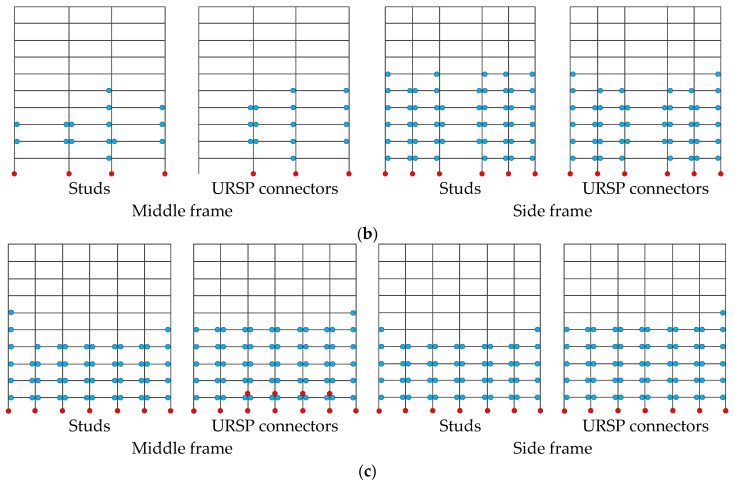
Distribution and failure modes of plastic hinges: (**a**) case XL1; (**b**) case YL1; (**c**) case XL2. Red and blue circular solid points represent the column and beam plastic hinges, respectively.

The distribution of plastic hinges and their failure modes in the structure under the action of large earthquakes are shown in Figure 13. In case XL1, the distribution of plastic hinges of the middle frame and the side frame was different. For the middle frame, the degree of plasticity was more severe and the plasticity appeared at the bottom and the second-floor column bottom. For the side frame, plastic hinges appeared at the bottom of the bottom column, and only at the beam ends in the rest of the frame, in line with the strong column–weak beam design criteria. The figure shows that compared with traditional studs, the full-span URSP connection structure had more hinges, the overall degree of plasticity of the structure was more severe, and the structure was more unfavorable for earthquake resistance.

In case YL1, plastic hinges only appeared at the bottom of the bottom columns and beam ends for the middle frame and side frame. The figure illustrates that the overall degree of plasticity of the full-span URSP connection was less severe than that of traditional stud connection structures and that the number of plastic hinges of bottom columns and beams was lesser, which was more favorable for earthquake resistance.

In case XL2, the distribution of plastic hinges in the middle and side frames was different. For the middle frame, plastic hinges appeared at the bottom of the bottom columns and at the beam ends in other parts. After the full-span URSP connection was arranged, plastic hinges also appeared in the second-floor columns; meanwhile, the overall plasticity was more severe, and the seismic resistance was more unfavorable. For the side frame, the traditional stud and the full-span URSP connection structures only had column plastic hinges at the bottom, while other plastic hinges occurred at the beam ends. However, the overall plasticity of the full-span URSP structure was more severe than that of the traditional stud connection structure. The full-span URSP connection demonstrated a more adverse result for the seismic safety of the structure.

Based on the above analysis of the development of plastic hinges under the action of large earthquakes within several calculation conditions, there was no clear conclusion regarding whether the full-span URSP connection was more favorable than the traditional stud connection. Nevertheless, the influence of the full-span URSP connectors on plastic hinge development was not significant. When the half-span URSP connection arrangement was adopted, since the response difference of each floor under seismic loads compared with the traditional stud connection was slight, the overall plastic development degree barely changed.

**Figure 14:**
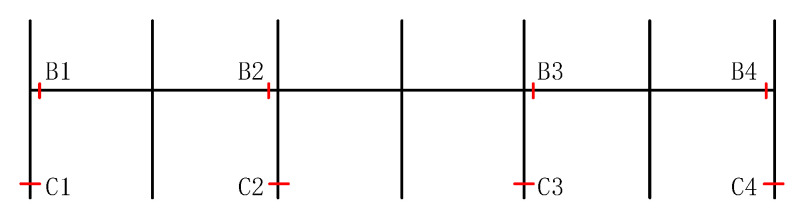
Numbering of key sections.

**Figure 15:**
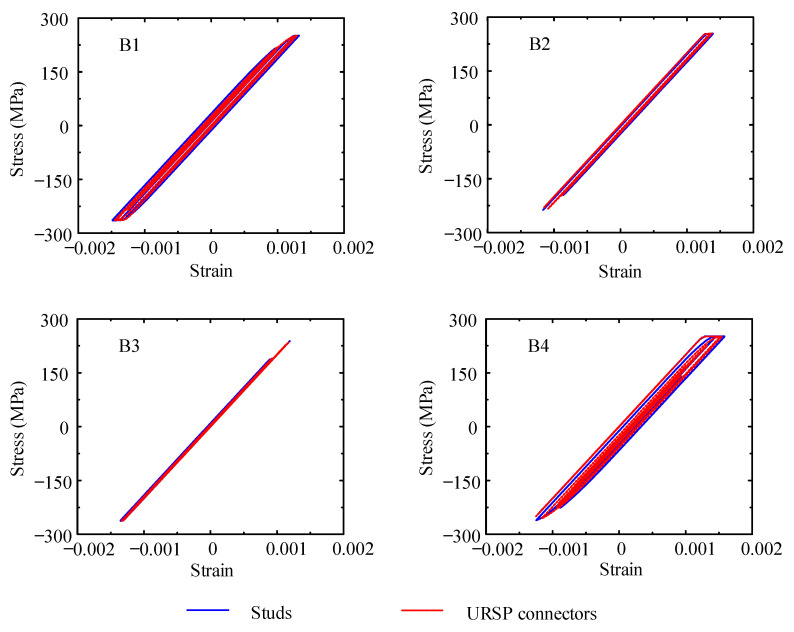
Stress–strain curves of steel of the beam section of the 1st floor.

**Figure 16:**
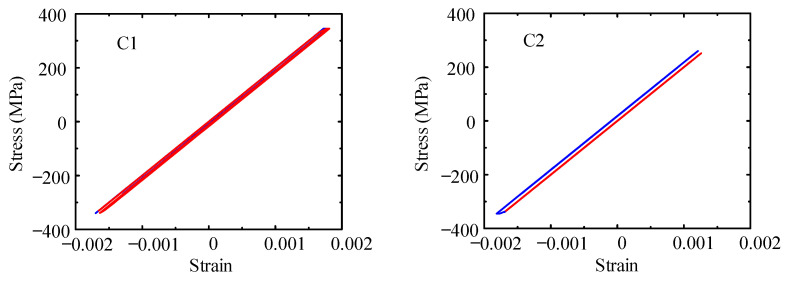

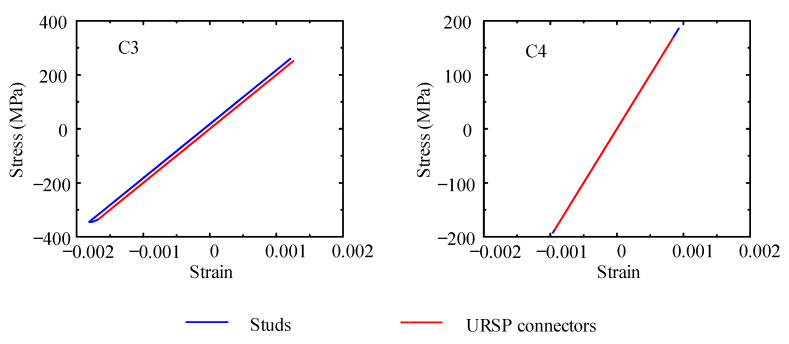
Stress–strain curves of steel of the bottom column.

To further explore the influence of URSP connectors, the stress distribution and failure characteristics of the cross-section behavior of key beams and columns of the bottom floors were analyzed and compared with those of traditional stud connections. Figure 14 depicts the numbering of key sections, including beam sections B1–B4 on the first floor and column base sections C1–C4 on the bottom column. Figure 15 and Figure 16 show the stress–strain relationship curves of the edge steel plates of beam sections B1–B4 and column base sections C1–C4, respectively, under seismic wave 1. Similar conclusions can be drawn from these figures in that the difference in the stiffness and plastic development between full-span URSP connectors and studs was ignorable.

### 4.4. Discussion

The results in this section show that half-span URSP connectors had little influence on the seismic performance of the composite frame system in terms of roof displacement, inter-story displacement, and the distribution and failure modes of plastic hinges. However, the effect of the full-span arrangement of URSP connectors was complex and diverse in different cases. Previous static and pseudo-static test studies on the application of URSP connectors found that a partial arrangement rarely affects the capacity and behavior of structures while a full-span arrangement decreases the stiffness of structures [32,36,37]. These results are consistent with the dynamic results presented in this paper. Future studies will be conducted to consider more cases to further support these findings.

## 5. Conclusions

In this study, beam–shell mixed finite element models were established to investigate the seismic response of steel–concrete composite frame structures with URSP connectors. The feasibility of the proposed model was proven by frame test results. The results of structures with URSP connection were compared with the results of structures with a traditional stud connection through model analysis and time history analysis under several seismic waves. The main conclusions are as follows:

(1) The results of the horizontal seismic elasto–plastic time history analysis revealed that the full-span arrangement of URSP connectors under ultimate conditions had little effect on the first few natural vibration periods of the composite frame structure. However, it had different effects on seismic performance under different seismic loads. Compared with the results of the traditional stud connection, the calculated values of roof lateral displacement and inter-story drift angle were slightly larger in most cases. Specifically, this arrangement increased the maximum inter-story displacement by 31.5% and induced adverse effects in certain cases. In addition, the slight differences observed in the positions of plastic hinges, failure mechanism, and degree of plasticity development of the structure may result in adverse effects on earthquake resistance. In practical engineering, it is not recommended to adopt a full-span arrangement of URSP connectors.
(2) In comparison with the traditional stud connection arrangement, the arrangement of half-span URSP connectors had little influence on the seismic performance of the composite frame structure, which still showed the good seismic performance of the original composite structure. The composite frame with a half-span arrangement can still be designed according to the relevant specifications of the traditional connection composite frame.
(3) When URSP connectors are partially arranged, one should still consider the effect of the spatial composite effect of the floor on the composite frame under seismic actions, which is the same as that of a traditional stud connection composite frame.

This paper found that the arrangement of half-span URSP connectors had little influence on the seismic performance of the composite frame system, and the obtained numerical results were based on four different seismic cases and a regular 10-story steel–concrete composite frame. System-level experimental and numerical studies considering more cases should be conducted to further support these findings. These further studies will help to promote the application of URSP connectors in composite frame systems.

## Figures and Tables

**Table 1:** Natural vibration characteristics with various interface connections.

Mode	Natural Vibration Period (s)					Ratio (Full-Span URSP/Full-Span Stud)	Mode Characteristic
	Common Nodes	Full-Span Stud	Full-Span URSP	Spring Stiffness = 0	Steel Beam		
1	1.000	1.218	1.220	1.277	1.533	1.002	Y-translation
2	0.943	1.116	1.119	1.182	1.399	1.003	X-translation
3	0.799	0.923	0.926	0.970	1.154	1.003	Z-rotation
4	0.329	0.397	0.403	0.569	0.498	1.015	Y-translation
5	0.311	0.366	0.374	0.556	0.457	1.022	X-translation
6	0.265	0.305	0.311	0.484	0.378	1.020	Z-rotation
7	0.191	0.227	0.238	0.481	0.282	1.048	Y-translation
8	0.181	0.211	0.224	0.465	0.261	1.062	X-translation
9	0.155	0.189	0.189	0.458	0.218	1.000	Z-rotation

**Table 2:** Ratios of the maximum roof displacements.

Case XL1	Case YL1	Case YS1		Case XL2
$\frac{D_{U}}{D_{S}}$	$\frac{D_{U}}{D_{S}}$	$\frac{D_{U}}{D_{S}}$	$\frac{D_{U-h}}{D_{S}}$	$\frac{D_{U}}{D_{S}}$
1.043	1.031	1.183	1	1.128

Note: *D_U_*, *D_S_*, and *D_U−h_* are the maximum roof displacements of full-span URSP, full-span studs, and half-span URSP, respectively.

**Table 3:** The maximum inter-story displacements (unit: mm).

Floor	Case XL1		Case YL1		Case YS1		Case XL2	
	$d_{U}$	$d_{S}$	$d_{U}$	$d_{S}$	$d_{U-h}$	$d_{S}$	$d_{U}$	$d_{S}$
10	17.9	15	13.5	14	3.5	2.7	14.5	12.4
9	24	20.5	22	23.1	5.6	4.4	23.3	21.2
8	34.4	27.4	30.9	32.2	7.9	6.3	31.4	28.9
7	36.8	34.5	39.3	39.6	10.0	8.1	36.7	33.8
6	41.2	41.3	46.7	45.1	11.5	9.5	40.6	35.4
5	46.6	47.7	54.5	51.5	12.8	10.8	45.1	38.2
4	51.5	53.7	60.3	57.3	13.8	11.9	48	44.6
3	54.5	55.2	61.6	60.1	14.2	12.4	51.7	48.8
2	49	47.6	53.3	53.3	13.2	11.6	46.3	46.1
1	24.1	21.4	30.7	30.1	7.9	6.7	26.8	27.5

Note: *d_U_*, *d_S_*, and *d_U_*_−*h*_ are the maximum inter-story displacements of full-span URSP, full-span studs, and half-span URSP, respectively.

**Table 4:** The ratios of the maximum inter-story displacements (unit: mm).

Floor	Case XL1	Case YL1	Case YS1		Case XL2
	$\frac{d_{U}}{d_{S}}$	$\frac{d_{U}}{d_{S}}$	$\frac{d_{U}}{d_{S}}$	$\frac{d_{U-h}}{d_{S}}$	$\frac{d_{U}}{d_{S}}$
10	1.188	0.967	1.315	1.002	1.166
9	1.167	0.951	1.288	0.997	1.097
8	1.111	0.960	1.260	0.997	1.088
7	1.066	0.991	1.232	1.000	1.086
6	0.998	1.034	1.201	1.001	1.146
5	0.977	1.058	1.181	1.000	1.182
4	0.959	1.052	1.166	1.002	1.075
3	0.987	1.026	1.150	1.002	1.061
2	1.030	1.000	1.145	1.007	1.005
1	1.129	1.020	1.170	1.019	0.973

Note: *d_U_*, *d_S_*, and *d_U_*_−*h*_ are the maximum inter-story displacements of full-span URSP, full-span studs, and half-span URSP, respectively.
